# Trends in oncological imaging during the COVID‐19 pandemic through the vaccination era

**DOI:** 10.1002/cam4.5678

**Published:** 2023-02-12

**Authors:** Debby Cheng, Soham Ghoshal, Ottavia Zattra, Moses Flash, Min Lang, Raymond Liu, Michael H. Lev, Joshua A. Hirsch, Sanjay Saini, Michael S. Gee, Marc D. Succi

**Affiliations:** ^1^ Harvard Medical School Boston Massachusetts USA; ^2^ Department of Radiology Massachusetts General Hospital Boston Massachusetts USA; ^3^ Medically Engineered Solutions in Healthcare Incubator, Innovation in Operations Research Center (MESH IO) Massachusetts General Hospital Boston Massachusetts USA

**Keywords:** cancer, computed tomography, COVID‐19 pandemic, imaging

## Abstract

**Background:**

This study examines the impact that the COVID‐19 pandemic has had on computed tomography (CT)‐based oncologic imaging utilization.

**Methods:**

We retrospectively analyzed cancer‐related CT scans during four time periods: pre‐COVID (1/5/20–3/14/20), COVID peak (3/15/20–5/2/20), post‐COVID peak (5/3/20–12/19/20), and vaccination period (12/20/20–10/30/21). We analyzed CTs by imaging indication, setting, and hospital type. Using percentage decrease computation and Student's *t‐*test, we calculated the change in mean number of weekly cancer‐related CTs for all periods compared to the baseline pre‐COVID period. This study was performed at a single academic medical center and three affiliated hospitals.

**Results:**

During the COVID peak, mean CTs decreased (−43.0%, *p* < 0.001), with CTs for (1) cancer screening, (2) initial workup, (3) cancer follow‐up, and (4) scheduled surveillance of previously treated cancer dropping by 81.8%, 56.3%, 31.7%, and 45.8%, respectively (*p* < 0.001). During the post‐COVID peak period, cancer screenings and initial workup CTs did not return to prepandemic imaging volumes (−11.4%, *p* = 0.028; −20.9%, *p* = 0.024). The ED saw increases in weekly CTs compared to prepandemic levels (+31.9%, *p* = 0.008), driven by increases in cancer follow‐up CTs (+56.3%, *p* < 0.001). In the vaccination period, cancer screening CTs did not recover to baseline (−13.5%, *p* = 0.002) and initial cancer workup CTs doubled (+100.0%, *p* < 0.001). The ED experienced increased cancer‐related CTs (+75.9%, *p* < 0.001), driven by cancer follow‐up CTs (+143.2%, *p* < 0.001) and initial workups (+46.9%, *p* = 0.007).

**Conclusions and relevance:**

The pandemic continues to impact cancer care. We observed significant declines in cancer screening CTs through the end of 2021. Concurrently, we observed a 2× increase in initial cancer workup CTs and a 2.4× increase in cancer follow‐up CTs in the ED during the vaccination period, suggesting a boom of new cancers and more cancer examinations associated with emergency level acute care.

## Graphical abstract

The pandemic continues to impact cancer care. We observe significant declines in cancer screening CTs through the end of 2021. Concurrently, we observe a 2x increase in initial cancer workup CTs and a 2.4x increase in active cancer CTs in the ED during a time period following the introduction of vaccinations (20th December 2020‐30th October 2021), suggesting a boom of new cancers and more cancer exams associated with emergency level acute care.

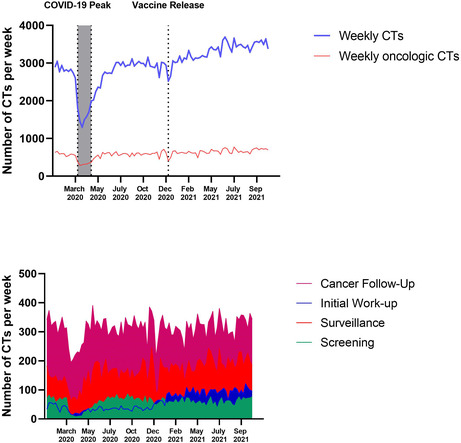

## 1 INTRODUCTION

### 1.1 Background

The COVID‐19 pandemic introduced major challenges to the care of cancer patients. Initial responses to the pandemic focused on deferring routine care.^1^, ^2^, ^3^ Cancer care sites rescheduled screenings, diagnostic imaging, and surgeries in an effort to conserve limited hospital resources.^4^ Additionally, oncology patients may be at increased risk for serious illness from COVID‐19 because of immunosuppression caused both by their disease and by the therapies they receive.^5^, ^6^, ^7^

Not only did medical centers delay oncologic care, but patients also chose to defer treatment. In a longitudinal study surveying the COVID‐19 pandemic's impact on cancer patients, 47% of 1199 respondents indicated that they had delayed care.^8^ Fear of COVID‐19 among respondents was correlated with higher percentages of delaying care, a trend that persisted throughout the pandemic.^8^ Furthermore, financial strain imposed by the COVID‐19 pandemic affected oncology patients' ability to afford medical care.^9^ By July 2020, cancer centers began resuming routine care, while taking measures to protect patients and healthcare workers.^10^, ^11^

Imaging was one of the aspects of cancer care that was severely impacted by the pandemic.^12^, ^13^, ^14^, ^15^, ^16^, ^17^, ^18^ One study found that after “nonessential” service closure in March 2020, weekly imaging volume declined by 54% at its large urban academic hospital and 64% at affiliated imaging centers.^19^ Other studies have also reported significant volume declines in 2020 across various imaging modalities, including mammography, nuclear medicine, MRI, ultrasound, interventional, CT, and radiography.^19^, ^20^, ^21^

Previously published research showed that CTs for cancer screenings and initial workups at our institution did not return to prepandemic levels through the end of 2020.^22^ Moreover, there was redistribution of oncologic CT imaging utilization away from outpatient settings, toward ED‐ and inpatient‐based care.^22^ The present study investigates how these trends changed beyond 2020 and into the 2021 postvaccination era. This information may provide insight into the pandemic's long‐term repercussions on cancer imaging care as well as inform institutional focus for this care population.

### 1.2 Purpose

The study investigates trends in the utilization of cancer‐related CTs at an academic hospital and affiliated hospitals between January 2020 and October 2021.

## 2 METHODS

### 2.1 Study Design

This received approval with exemption from our Institutional Review Board (IRB) and was compliant with the Health Insurance Portability and Accountability Act. This was a retrospective time series analysis of all CT scans performed between 5 January 2020 and 30 October 2021 at one large academic institution and three affiliated community medical centers. This study uses methodology similar to that of our previous publication^22^ to analyze, for the first time, CT trends that extend into the 2021 postvaccination era.

### 2.2 Setting

The main hospital is an urban quaternary academic center (QAC) with 1017 beds treating approximately 50,000 inpatients, 1.5 million outpatients, and 100,000 ED patients annually. Our three affiliated community centers are a 273‐bed suburban university‐affiliated community hospital (UACH), a 25‐bed sole community hospital (SCH), and a 19‐bed sole community hospital (SCH). The analysis pooled data from the two SCHs into one hospital setting category.

### 2.3 Data Extraction and Processing

**FIGURE 1:**
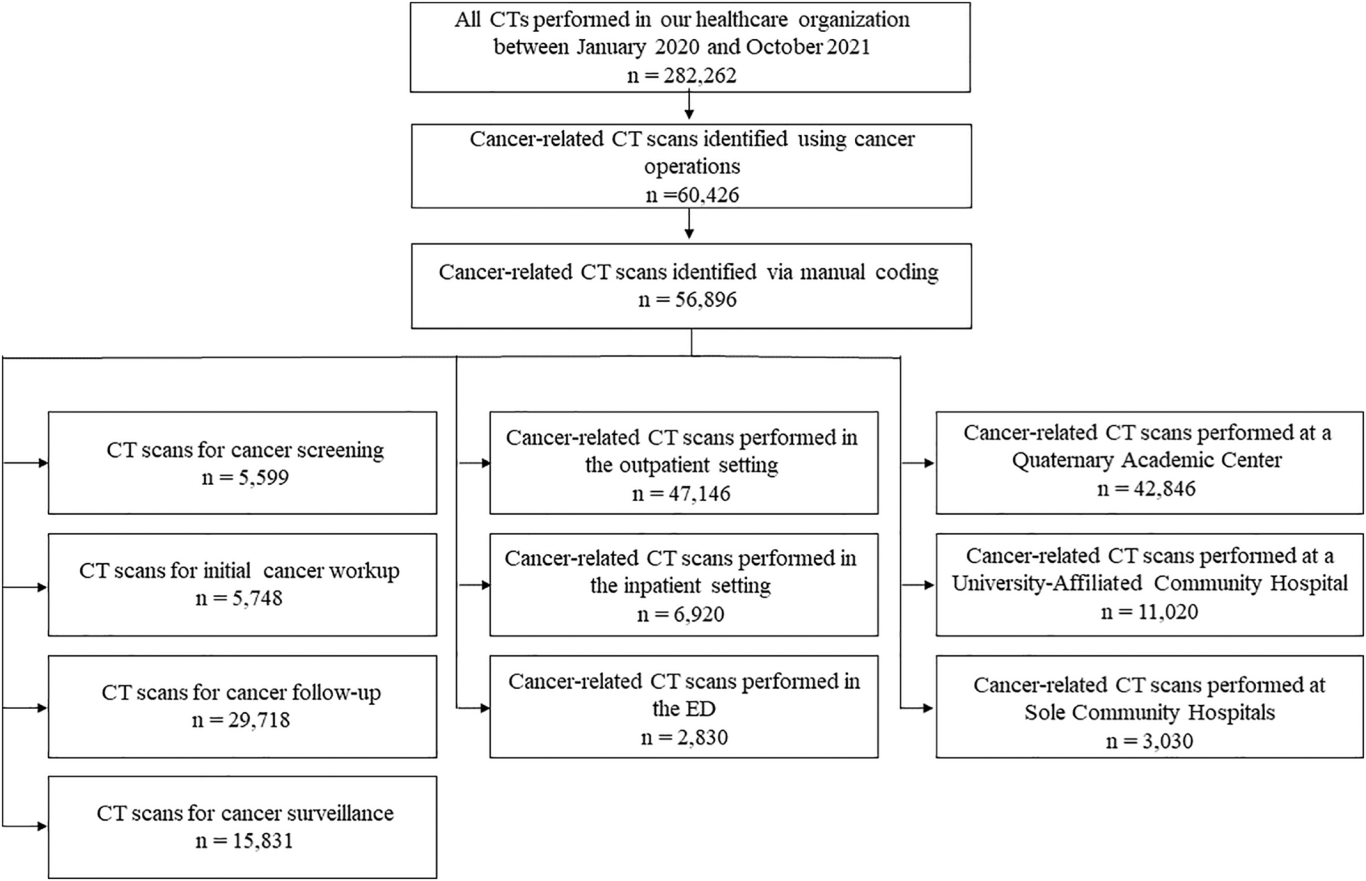
Data analysis algorithm.

Data were extracted from our electronic medical record (Epic Systems, Verona WI). Data included the number of individual CT scans performed during the 22‐month study period, across all four hospitals (1 QAC, 1 UACH, 2 SCHs), yielding 284,756 CT examinations. We adapted our previously published methodology and identified potential oncological CT scans by conducting a structured search of cancer‐related terms as listed in Table 1.^22^ CT scan indications were then (1) manually screened by a blinded board‐certified radiologist and a radiology fellow to ensure accuracy, (2) categorized by hospital setting and care setting (Figure 1), and (3) manually coded by a blinded board‐certified radiologist and a radiology fellow into four imaging indication categories based on the stage of cancer care: (i) cancer screening (ii) initial workup of new suspected cancer or new cancer rule‐out; (iii) cancer follow‐up (i.e., imaging for patients with confirmed active cancer); and (iv) scheduled surveillance of patients with no active cancer but with a history of previously treated cancer.

**TABLE 1:** Cancer‐related terminology used for structured search of the order requisition field of CT imaging examinations.

Search term
Malignant Neoplasm Metastasis Mets Metastatic Surveillance Staging Malignancy Cancer Mass Lump Tumor Tumor ‐oma Malig

The following comparison periods were defined using state‐wide guidelines and legislation: pre‐COVID‐19 (5 January 2020–14 March 2020), COVID‐19 peak (15 March 2020–2 May 2020), post‐COVID‐19 peak (3 May 2020–19 December 2020), and postvaccine release (20 December 2020‐30 October 2021).^23^, ^24^ Each weekly time period began on Sunday and ended on Saturday. We considered the end of the pre‐COVID‐19 period to be the week of 10 March 2020 because this was the date our State declared a state of emergency. The week of 3 May 2020 was considered the beginning of the post‐COVID‐19 peak because this was the day that nonurgent imaging was reimplemented at our institution after the initial lockdown. Finally, we considered the week of 15 December 2020 (a weekday) to be the end of the post‐COVID‐19 peak as this was the beginning of the vaccine distribution in our State—which prioritized healthcare workers, front‐line workers, and vulnerable populations. We began analysis of the “post‐vaccine release” period on Sunday, 20 December 2020. We considered the postvaccine release period to start at the first day of the vaccine distribution plan rather than when the general population became eligible for vaccinations because this initial vaccine distribution phase had implications for expanding hospital workflow and scheduling.

### 2.4 Statistical methods

For each of the four study periods, we calculated mean weekly CTs. The pre‐COVID‐19 period represented weeks 1–10, the COVID‐19 peak represented weeks 11–17, the post‐COVID‐19 peak represented weeks 18–50, and the postvaccine release period represented weeks 51–95. The pre‐COVID‐19 period was designated the prepandemic baseline number of oncological CTs. Percentage decrease computation and Student's two‐sample *t‐*test were used to compare all time periods to the prepandemic baseline period across examination indication, care setting, and hospital type using GraphPad Prism (GraphPad, San Diego, CA) and Excel (Microsoft, Redmond, WA).

## 3 RESULTS

### 3.1 COVID‐19 peak (15 March 2020–2 May 2020)

**FIGURE 2:**
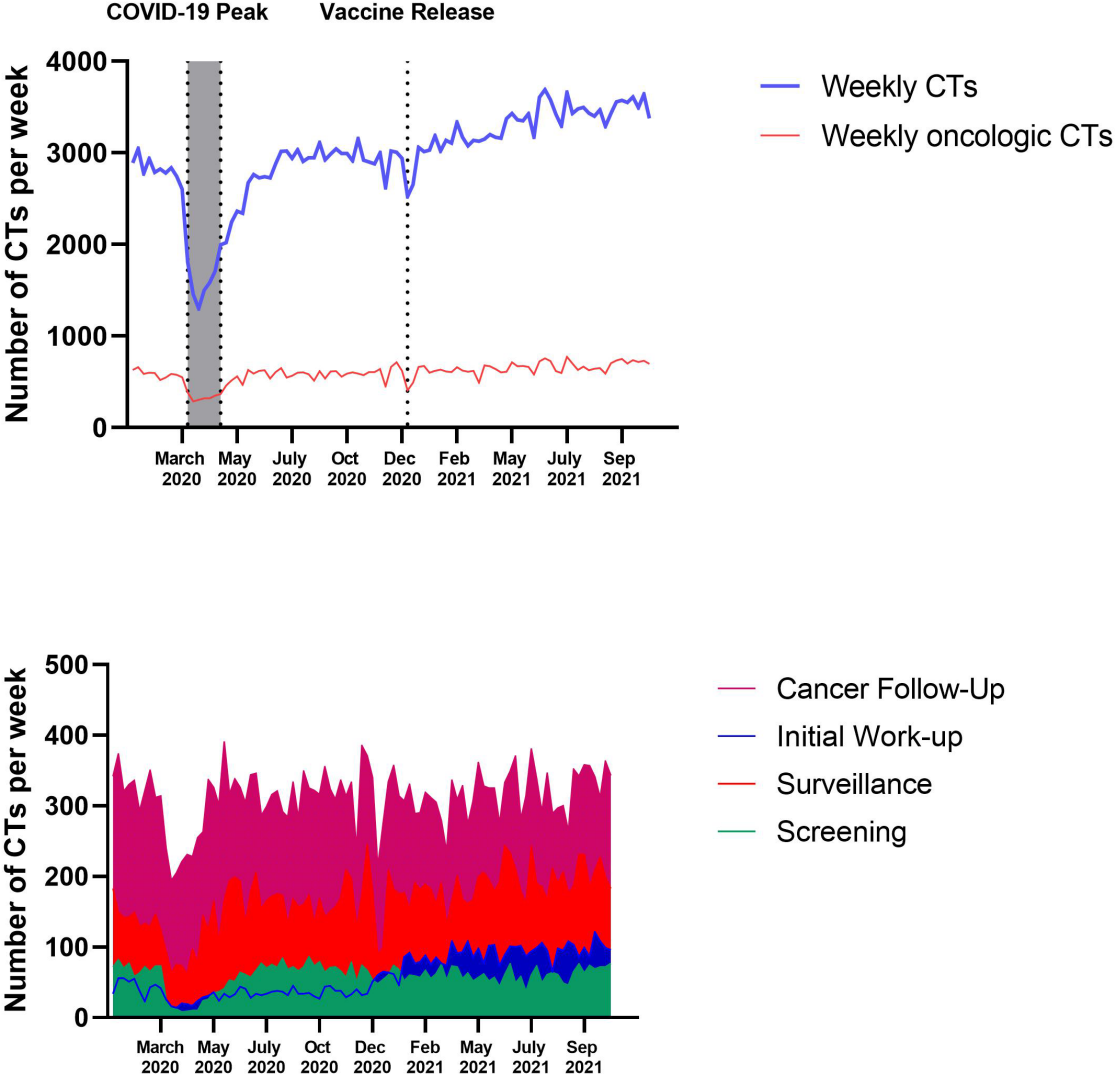
Average number of all CTs and oncologic CTs per week (top) and average number of CTs per week by imaging indication (bottom) from 5 January 2020 to 30 October 2021 (a 95‐week period).

On 10 March 2020, our State entered a State of Emergency resulting in a decrease in number of cancer‐related CTs from a prepandemic weekly average of 587 CTs to 334 CTs during the pandemic peak (−43.0%; *p* < 0.001; Table 2; Figure 2). This negative trend in the number of cancer‐related CTs mirrored that of all CTs at our institution (−42.5%, *p* < 0.001), and the rate of decline of cancer‐related CTs matched that of all CTs. This indicates that the proportion of oncological CT scans remained relatively constant (pre‐COVID‐19 vs. COVID‐19 peak: 20.8% vs. 20.6%).

**TABLE 2:** The number of cancer‐related computed tomography (CT) scans as compared to all CT scans by imaging indication, hospital type, and care setting during four periods in 2020–2021. All statistics compare mean frequency of CT scans.

	Pre‐COVID‐19 peak (baseline CTs per week) (Mean ± SD	COVID‐19 peak (CTs per week) (Mean ± SD)	Percent of baseline	*p* Value^a^	Post‐COVID‐19 peak (CTs per week) (Mean ± SD)	Percent of baseline	*p* Value^a^	(CTs per week) (Mean	Percent of Baseline	*p* Value^a^
All CTs										
All CTs	2823.00 ± 118.70	1623.71 ± 234.62	57.5	<0.001	2840.76 ± 259.83	100.6	0.764	3309.36 ± 249.02	117.2	<0.001
Oncologic CTs	586.60 ± 40.08	334.43 ± 35.01	57.0	<0.001	585.52 ± 57.94	99.8	0.947	652.60 ± 72.01	111.3	<0.001
Imaging indication										
Cancer screening	70.50 ± 6.95	12.86 ± 6.23	18.2	<0.001	62.45 ± 15.73	88.6	0.028	60.96 ± 9.84	86.5	0.002
Initial workup	44.50 ± 10.78	19.43 ± 4.31	43.7	<0.001	35.21 ± 5.96	79.1	0.024	89.00 ± 15.71	200.0	<0.001
Cancer follow‐up	329.00 ± 22.67	224.86 ± 20.60	68.4	<0.001	321.73 ± 32.10	97.8	0.433	316.38 ± 35.69	96.2	0.173
Surveillance	142.60 ± 16.78	77.29 ± 13.91	54.2	<0.001	166.12 ± 28.33	116.5	0.003	186.3 ± 31.24	130.6	<0.001
Care setting										
Outpatient	499.90 ± 40.79	280.43 ± 30.87	56.1	<0.001	481.36 ± 55.21	96.29	0.263	540.0 ± 65.10	108.0	0.022
Inpatient	66.30 ± 9.71	36.43 ± 5.77	55.0	<0.001	77.24 ± 11.19	116.5	0.008	76.73 ± 12.55	115.7	0.010
ED	20.40 ± 5.95	17.57 ± 3.69	86.1	0.246	26.91 ± 5.92	131.9	0.008	35.89 ± 6.51	175.9	<0.001
Hospital setting										
QAC^b^	457.00 ± 38.70	267.86 ± 29.08	58.6	<0.001	433.73 ± 40.97	94.9	0.120	490.84 ± 59.83	107.4	0.037
UACH^c^	102.4 ± 8.92	52.57 ± 10.88	51.3	<0.001	119.33 ± 20.99	116.5	<0.001	126.44 ± 19.39	123.5	<0.001
SCHs^d^	27.20 ± 8.34	14.00 ± 6.00	51.5	0.002	32.45 ± 7.86	119.3	0.099	35.31 ± 7.31	129.8	0.015

Grey shaed reflect COVID‐19 periods: White = Pre‐COVID‐19; Light Grey = COVID‐19 peak; Medium Grey = Post‐COVID‐19; Dark Grey = Postvaccine.

^a^ Compared to pre‐COVID‐19 peak period.

^b^ Quaternary academic center.

^c^ University‐affiliated community hospital.

^d^ Sole community hospitals.

**FIGURE 3:**
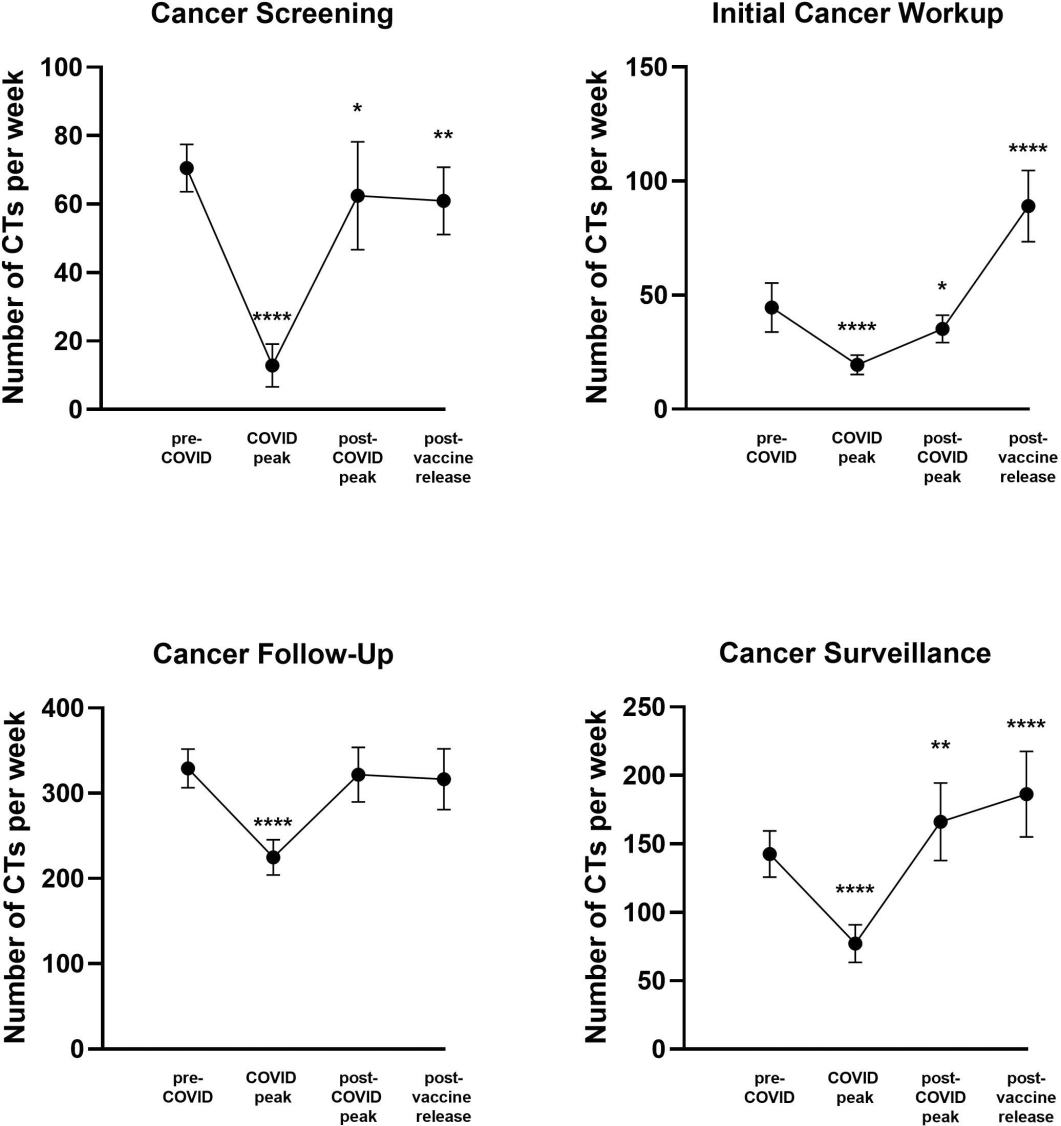
Average number of computed tomography scans per week by imaging indication across the three study periods. * < 0.05, ** < 0.01, *** < 0.001, **** < 0.0001.

**FIGURE 4:**
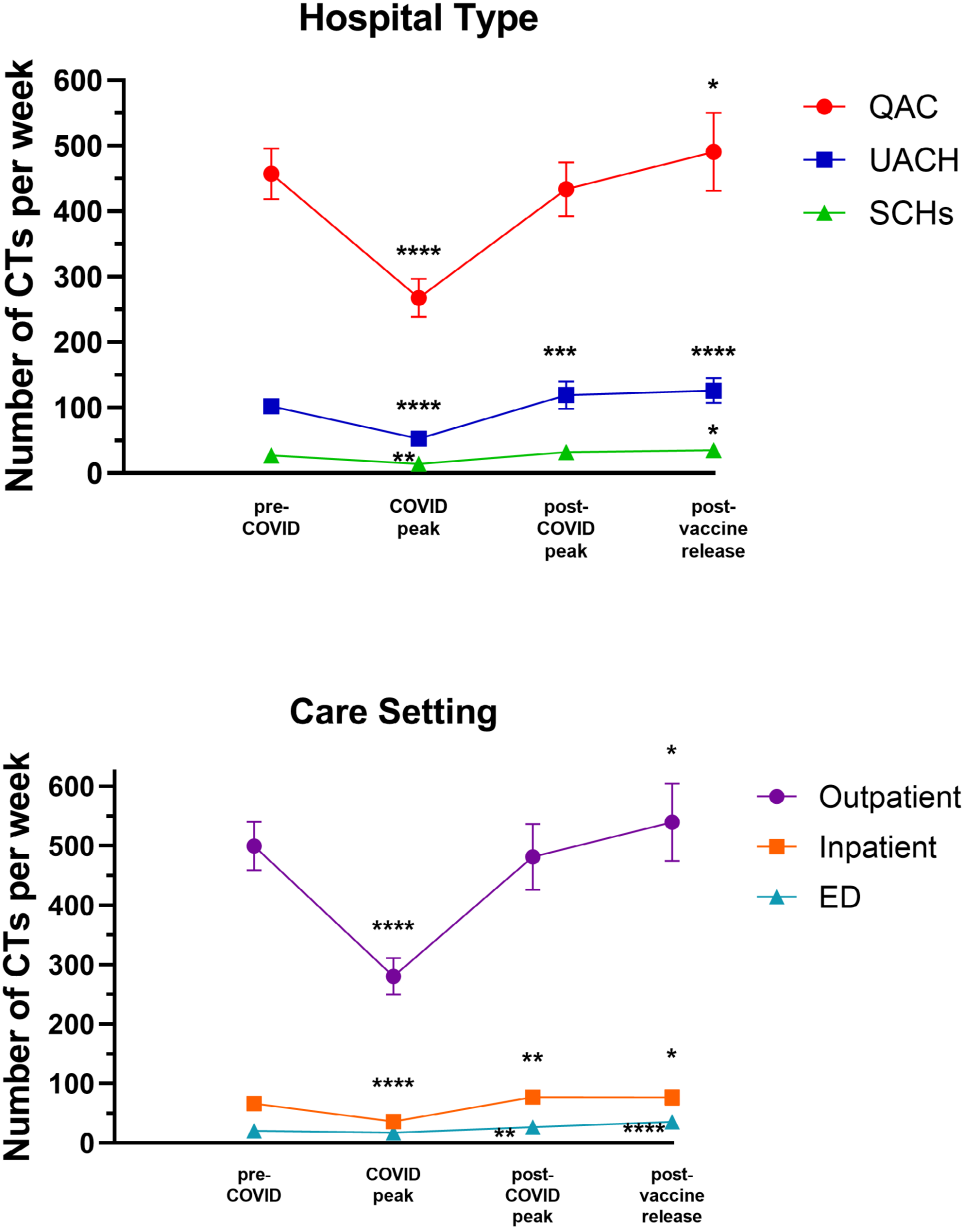
Average number of computed tomography scans per week by hospital type and care setting across the three study periods. * < 0.05, ** < 0.01, *** < 0.001, **** < 0.0001.

The number of CTs conducted weekly for all stages of cancer care indications experienced a significant decline during the peak of the pandemic. Compared to baseline, cancer screening CTs decreased by 81.8% (*p* < 0.001), initial workup CTs decreased by 56.3% (*p* < 0.001), cancer follow‐up CTs decreased by 31.7% (*p* < 0.001), and surveillance scans decreased by 45.8% (*p* < 0.001, Figure 3). With regard to care settings, the number of weekly CTs completed in outpatient and inpatient settings declined significantly during the peak of the pandemic (−43.9%, *p* < 0.001; −45.1%, *p* < 0.001, respectively), but the number of CTs conducted in the ED did not significantly change compared to baseline (−13.9%, *p* = 0.246, Figure 4). All hospital types experienced declines in cancer CTs. QACs experienced a 41.4% decline (*p* < 0.001), UACHs experienced a 48.7% decline (*p* < 0.001), and SCHs experienced a 48.5% decline (*p* < 0.01, Figure 4).

### 3.2 Postpeak period (3 May 2020–19 December 2020)

During the postpeak period, there was differential recovery of oncological CTs across imaging indication, care setting, and hospital type.

Weekly mean CTs recovered to baseline for cancer follow‐ups (−2.2%, *p =* 0.433) and rebounded above baseline for surveillance scans (+16.5%, *p =* 0.003). Weekly mean CTs for cancer screenings and initial workups did not return to baseline in the postpeak period (−11.4%, *p* = 0.028; −20.9%, *p* = 0.024, respectively, Figure 3). Across care settings, both the ED and inpatient settings experienced increases in weekly mean CTs compared to baseline (+31.9%, *p* = 0.008; +16.5%, *p* = 0.008). The increase in weekly mean CTs in the ED was driven by an increase in the number of cancer follow‐up‐related CTs compared to baseline (+56.3%, *p* < 0.001), whereas no specific imaging indication drove the increase in CTs in inpatient settings. Outpatient weekly mean CTs recovered to baseline (Figure 4).

Across hospital types, the number of weekly mean CTs recovered to baseline for the QAC and SCHs, whereas for the UACH, weekly mean CTs rebounded above baseline (+16.5%, *p* < 0.001, Figure 4). Increases in the weekly mean CTs at the UACH were driven by increases in cancer follow‐up CTs and surveillance scans compared to baseline (+43.0%, *p* < 0.001; +31.2%, *p* = 0.029).

### 3.3 Postvaccine release period (20 December 2020‐30 October 2021)

The first phase of our state's COVID‐19 vaccine distribution plan began on 15 December 2020. During the months following, the weekly volume of cancer CTs at our institution increased above baseline (+11.3%, *p* < 0.001, Figure 2).

An increase in the weekly mean CTs was observed across all care settings (outpatient = +8.0%, *p* = 0.022; inpatient = +15.7%, *p* = 0.010; ED = +75.9%, *p* < 0.001) and hospital types (QAC = +7.4%, *p* = 0.037; UACH = +23.5%, *p* < 0.001; SCHs = +29.8%, *p* = 0.015, Figure 4). Across imaging indications, the weekly mean CTs recovered to baseline for cancer follow‐ups (−3.8%, *p* = 0.173) and rebounded above baseline for initial workups (+100.0%, *p* < 0.001) as well as surveillance scans (+30.6%, *p* < 0.001, Figure 3). Weekly mean CTs remained significantly below baseline for cancer screenings (−13.5%, *p* = 0.002). Cancer follow‐up and initial workup CTs increased in the ED (+143.2%, *p* < 0.001; +46.9%, *p* = 0.007) and drove the rebound in the number of cancer CTs within the ED above baseline.

## 4 DISCUSSION

The present study provides data on how COVID‐19 affected the delivery of cancer imaging care.^19^, ^22^, ^25^ Following the March–May COVID‐19 peak in 2020 (the postpeak period), weekly cancer‐related CTs for cancer screenings declined by 11.4% (*p* = 0.028) and the weekly cancer‐related CTs in the ED significantly increased by 31.9% (*p* = 0.008), a trend that extended into the end of 2021. In the period between December 2020 and October 2021, when mass vaccinations were introduced, weekly cancer‐related CTs for initial cancer workups increased significantly by 100.0% (*p* < 0.001), suggesting in‐part consequences of deferred care from the COVID‐19 peak among other factors.

Our results support previous studies in the United States that suggest decreases in public interest in cancer screening^26^ and that report declines in lung cancer screening CTs as well as other screening modalities, such as mammograms and colonoscopies, during the pandemic.^16^, ^22^, ^27^, ^28^ However, for the first time, there is evidence of the effects of delayed cancer care—a 2x increase in weekly initial cancer workups by CT and a 2.4× increase in weekly cancer follow‐up CTs in the ED. To our knowledge, no existing study has investigated the impact of the pandemic from its beginning through the end of 2021. The present study fills this gap in the literature by examining 18 months of recovery following the initial COVID‐19 peak.

During the peak of the COVID‐19 pandemic, there were significant declines in the weekly cancer‐related CTs for all stages of cancer care. Cancer screenings and initial workup CTs decreased by more than 50%. In the post‐COVID‐19 peak period between May and December 2020, weekly cancer‐related CTs returned to baseline levels, albeit not equally across all groups. Whereas the weekly cancer‐related CTs recovered to baseline for cancer follow‐ups and increased above baseline for surveillance scans, the number of cancer screenings and initial workup CTs remained significantly below baseline by 11.4% and 20.9%, respectively. Despite mass vaccination efforts, cancer screenings persistently and significantly remained below baseline by 13.5% and initial workup CTs doubled compared to pre‐COVID‐19 levels through the end of 2021.

Several factors may explain our findings. Although healthcare systems generally resumed imaging operations in early May 2020,^19^ in general, this requires ramp up‐periods that likely at least partially accounted for our findings of the postpeak period. Patients likely avoided seeking care due to concerns about acquiring the virus and lack of education about the importance of screening care. The lack of cancer screenings early in the pandemic may have caused a delay in the detection of cancer, as manifested in the significant increase of initial cancer workup and cancer follow‐up CTs in various care settings, such as the ED. Our findings that cancer‐related CT screening remained significantly below prepandemic levels well into the end of 2021 suggest further increases in initial workups in the coming months and years.

Importantly, after the initial COVID‐19 peak, the weekly number of oncological CTs in the ED increased above prepandemic levels, driven by increases in cancer follow‐up CTs during the postpeak period (+56.3%, *p* < 0.001) and by increases in both cancer follow‐up (+143.2%, *p* < 0.001) and initial workup CTs (+46.9%, *p* = 0.007) during the vaccination period. This finding suggests that the ED may be caring for more high‐acuity or advanced cancer cases with complications.^29^, ^30^, ^31^ Increases in cancer follow‐up CTs in the ED may reflect increases in medical emergencies among patients with known active cancers that may be attributed to pandemic‐related delays in care. Moreover, increases in initial cancer diagnoses in the ED may reflect delays in healthcare among undiagnosed cancer patients who now present in the ED with more acute symptoms that demand urgent treatment. This is consistent with studies that have found a rise in late‐stage cancer diagnoses after pandemic‐related declines in screenings.^32^, ^33^, ^34^

The high utilization of the ED for cancer follow‐up and initial cancer workup CTs suggests that patients are not receiving adequate cancer care. This may be due to pandemic‐related disruptions in cancer care, such as a backlog of cases, decreases in care capacity to accommodate social distancing, or diversion of resources toward COVID‐19 care. This potentially reversible ED utilization contributes to time‐ and cost‐inefficient cancer care.^29^, ^35^ As the backlog of cases reduces and hospitals increase their care capacity and resources, it is possible that this ED utilization will decrease to prepandemic levels.

This study has three main limitations. First, it is focused on CT imaging as it reflects consistent and widespread imaging in all stages and disease systems related to cancer care. Thus, major changes in the number of CTs are likely to mirror true changes in healthcare utilization. Even so, there are many other forms of cancer screening, such as biochemical testing and non‐CT based imaging (i.e., mammograms, MRI). We chose to exclude these due to their relatively limited disease‐specific application in cancer care. Second, we used the weekly mean CTs between January and March 2020 prior to the COVID‐19 peak as our baseline, which may not represent baseline imaging utilization from previous years. As with any single‐center analysis, generalizability is a limitation, although we did include several affiliated community hospitals to mitigate this.

Future studies should analyze (1) absolute numbers of new cancers being diagnosed in the ED, (2) the severity of newly diagnosed cancers, and (3) imaging indications as well as the patient diagnoses made following imaging. This would help verify the impact of substantial declines in cancer screening CTs throughout the pandemic.

In conclusion, the COVID‐19 pandemic severely impacted the use of CT imaging for cancer care in the United States. This study found significant declines in cancer imaging during the peak of the pandemic, followed by inconsistent recovery trends across imaging indications. Cancer screening CTs remained below prepandemic levels well into 2021, a finding that suggests that more patients with advanced cancers may present in the future. We also observed overall increases in the number of CTs within the ED that were driven by increases in cancer follow‐up and initial cancer workup CTs, a trend that suggests that the ED may be seeing more high‐acuity cancer cases. This study has implications for future medical practice because it highlights the importance of promoting cancer screening practices and avoiding the delay of cancer care. Whether through patient education, hospital policies, or public health initiatives, doing so both now as well as during future pandemics may allow us to catch cancers early, prevent crowding in the ED, reduce healthcare costs, and improve patient morbidity and mortality rates.

## AUTHOR CONTRIBUTIONS

**Debby Cheng:** Conceptualization (supporting); data curation (lead); formal analysis (lead); investigation (lead); methodology (lead); writing – original draft (lead); writing – review and editing (lead). **Soham Ghoshal:** Writing – review and editing (supporting). **Ottavia Zattra:** Formal analysis (supporting); methodology (supporting); writing – review and editing (supporting). **Moses Flash:** Formal analysis (supporting); methodology (supporting). **Min Lang:** Formal analysis (supporting); writing – review and editing (supporting). **Raymond Liu:** Writing – review and editing (supporting). **Michael H. Lev:** Writing – review and editing (supporting). **Joshua A. Hirsch:** Writing – review and editing (supporting). **Sanjay Saini:** Writing – review and editing (supporting). **Michael S. Gee:** Writing – review and editing (supporting). **Marc D. Succi:** Conceptualization (lead); data curation (supporting); formal analysis (supporting); investigation (supporting); methodology (supporting); writing – original draft (supporting); writing – review and editing (supporting).

## FUNDING INFORMATION

This research did not receive any specific grant from funding agencies in the public, commercial, or not‐for‐profit sectors

## CONFLICT OF INTEREST STATEMENT

The authors declare no relevant conflicts of interest.

## ETHICS APPROVAL

Approved with exemption by the Institutional Review Board.

## Data Availability

The data that support the findings of this study are available from the corresponding author upon reasonable request.
